# Cesarean section trends in Barrero, Dominican Republic: a community-level analysis of rates, risk factors, and patient motivations

**DOI:** 10.3389/fgwh.2026.1816133

**Published:** 2026-06-24

**Authors:** April Mabie, Alexandra Van Cleave, Olivia Anne Foley, Ryan Walters, Caron Gray, Jason Beste, Naomi Schmalz, Michelle María Jiménez de Tavárez

**Affiliations:** 1 Arrupe Global Scholars & Partnership Program, Creighton University, Omaha, NE, United States; 2 Department of Clinical Research and Public Health, Creighton University, Omaha, NE, United States; 3 Department of Obstetrics and Gynecology, Creighton University School of Medicine, Omaha, NE, United States; 4 Arrupe Global Scholars & Partnership Program, Creighton University, Phoenix, AZ, United States; 5 Department of Medical Education, Creighton University School of Medicine, Omaha, NE, United States; 6 Facultad de Ciencias de la Salud, Pontificia Universidad Catolica Madre y Maestra, Santiago de los Caballeros, Dominican Republic

**Keywords:** cesarean and vaginal deliveries, cesarean rate, Dominican Republic, insurance, motivation, private facilities, risk factor

## Abstract

**Introduction:**

This study, conducted in the Dominican community of Barrero, aimed to quantify rates of Cesarean sections (C-section) and explore their associations with age, education level, nationality, health insurance type, and delivery location. Additionally, this study aimed to uncover women's motivations for undergoing vaginal or C-section delivery for each birth.

**Methods:**

This cross-sectional study interviewed 121 women ages 18–50 years living in Barrero, Dominican Republic via house-to-house sampling in February 2025. Using a 39-question survey, information on all pregnancies for children 12 years and under was collected, yielding data for 236 pregnancies. Quantitative data was analyzed using generalized estimating equations to account for the correlation inherent to mothers providing responses on more than one pregnancy. Qualitative data was analyzed with thematic analysis using an inductive approach.

**Results:**

Of pregnancies measured, 71.2% were C-sections; 61.4% of these were described as planned (vs. emergent). Estimated C-section rate for the community of Barrero was 71.5%. Women were more likely to undergo Csection if they were older at the time of delivery (*p* = 0.008), had any level of education (*p* = 0.028), were Dominican compared to Haitian (77.2% vs. 9.3%, *p* = 0.001), had private insurance rather than either public or no insurance (82.9% vs. 65.0% vs. 56.5%, *p* = 0.004), and gave birth in a private hospital vs. a public hospital (84.5% vs. 61.9%, *p* = 0.013). The majority, 90.6%, of women delivering via C-section emergently cited “other” as their motivation while the majority of women delivering via vaginal birth cited personal preference. C-sections in Barrero, Dominican Republic are more likely in women who have any level of education, are of Dominican nationality, have private insurance, and deliver in a private hospital.

**Discussion:**

In Barrero, DR, C–section deliveries were found to have multifactorial associations. Csections were more common among women with any formal education, older maternal age, Dominican nationality, those who had private insurance, and those who delivered in private hospitals. Further studies must evaluate the influence healthcare institutions and providers have on women's decisions regarding C–section and interventions are needed to address this disparity in care.

## Introduction

Between 2000 and 2020, maternal mortality decreased globally, including within the Latin America and the Caribbean (LAC) region (1). However, the subregion of the Caribbean experienced an increase in maternal mortality during this 20-year span, making it one of two subregions globally where maternal mortality worsened (1). In the Caribbean, 1 in 260 women of reproductive age die of maternal causes, many of which are preventable (1). Of deaths from maternal causes, the subset *direct obstetric deaths* (or *direct maternal deaths*) include those related to complications from cesarean section (C-section) delivery (1).

Vaginal delivery is considered the safest birthing option for both mother and full-term fetus for its faster recovery, increased delivery safety, and lower infection rates when compared with C-section deliveries (2, 3). C-sections represent an operative form of delivery, introducing significant risks, including infection and post-partum hemorrhage (2, 3). Worldwide, hemorrhage is the number one cause of maternal death; infection is a significant contributor (4). Postpartum hemorrhage, defined as >1,000 mL blood loss during delivery, rates have been reported as 4.9% for unassisted vaginal deliveries, 8.5% for elective C-section, and 19.8% for non-elective C-section (5). Therefore, vaginal birth is the preferred delivery method and should be encouraged in prenatal counseling to prevent unnecessary death (6).

The rising rate of C-section deliveries constitutes a global crisis, in part due to medically unjustified operations (6). Since 1985, the World Health Organization (WHO) has recommended a desired C-section delivery rate of 10%–15%; however, in 2015 when the WHO convened to re-evaluate this recommendation, they stated there was not enough data to release a desired rate (7). More recently in 2023, the International Journal of Gynecology and Obstetrics asserted that the expected and desired rate for C-section deliveries globally is 20%–25% (6). In nations with a C-section rate approaching 70%, procedures are frequently performed for nonmedical indications, at maternal request, or for physician convenience (6). The C-section rate in the Dominican Republic (DR) has risen over the last 42 years, from close to global recommendations at 18.4% in 1983 to 62.9% in 2019, nearly triple the recommended rate (8, 9). Additionally, the World Bank reports maternal mortality in the DR has been increasing since 2000 from 76 deaths per 100,000 live births to 124 deaths per 100,000 live births in 2023 (10). Considering the correlation between morbidity/mortality and C-section deliveries, the C-section crisis in the DR must be addressed.

Several retrospective database studies have been conducted to identify factors associated with C-section deliveries in the DR (11–14). These studies identified positive correlations between higher rates of C-sections and greater wealth, delivery in a private birth institution, and greater education. Of note, Adu-Bonsaffoh et al. conducted a cross-sectional study in Ghana and the DR observing characteristics of women who received emergency C-sections and found positive correlations between emergent C-sections and nulliparous birth and birth with no history of C-section in both countries (15).

Previous research in the DR has clearly demonstrated that the national C-section rate is concerningly high and related to wealth, delivery in a private facility, and education status. However, national data from the DR often includes strictly urban or rural communities, leaving out many communities, such as Barrero. Barrero is a semi-rural, semi-urban community with an estimated population of 800 families. The majority of community members range in age from zero to 60 years old. No recent studies exist that quantify C-section rates based on insurance and prenatal care uptake, or further elaborate on women's perceptions, influences, and motivations for having C-sections in the Barrero community. Creating a database of this information from this community is important to develop a better understanding of birth experiences in previously unstudied regions and community types in the DR. This study aimed to quantify the C-section and vaginal delivery rates of adult women with children 0–12 years old in the Barrero community and evaluate correlations between delivery type (C-section or vaginal) and age, education level, nationality, health insurance status, type of birthing institution, and receipt of prenatal care. Further, this study worked to understand perceptions held by these women about their delivery.

## Materials and methods

This cross-sectional study used a mixed-methods design with a questionnaire developed by study authors AM, AV, and OF to identify the factors associated with C-section rates, quantify the utilization of prenatal care, and describe the delivery experiences of adult women of the Barrero community with children aged 0–12 years old. A questionnaire was chosen to offer closed-ended questions with open-ended response options to identify themes (16). The main topics investigated include demographic information, prenatal care access, risk factors, delivery type, motivations for preferred delivery type, and delivery locations.

### Questionnaire design

The questionnaire was developed after conducting a thorough literature review and incorporating relevant variables, specifically for satisfaction and perception about delivery (17–20). The study instrument included a paper quantitative questionnaire with 39 questions, some of which included open-ended “other” options. The questionnaire underwent editing and assessment of face validity by senior faculty at both Creighton University and Pontificia Universidad Católica Madre y Maestra (PUCMM). A pilot study was conducted by authors AM, AV, and OF for the questionnaire with peers and colleagues to understand flow, timing, and fatigue. Following this pilot study, edits were made to the study instrument to improve phrasing and remove redundant questions. The final questionnaire was translated to Spanish using the professional translation service, Cyracom International.

The initial section of the questionnaire pertained to demographic information and included respondent age, nationality, native language, insurance status at time of delivery, and education level. The second section pertained to birth-specific prenatal care information that included the existence and type of prenatal care received, and, if prenatal care was received, where it took place, the number of visits, when care started in the pregnancy, and details about birthing plan discussions that took place during prenatal visits. The third section pertained to birth-specific delivery method information and included items specific to delivery location, facility type, type of delivery, recovery time, motivation for delivery type, and most influential person in the selection of delivery type. In this section, women were asked whether their C-section was classified as emergent or planned.

### Recruitment and data collection

Inclusion criteria for participants included women of the Barrero community between the ages of 18 and 61 with children aged 0–12 years. This age range represents women who are legal adults who would also be considered fertile up to 12 years prior to the survey (World Health Organization, n.d.). In areas where electronic medical records are unavailable, maternal recall represents an important alternative, as women are accessible and have been shown to accurately and reliably report perinatal outcomes for up to 15 years after childbirth (21, 22). Interviewers AM, AV, and OF acquired age of participants and their children using maternal recall via direct questioning. Individuals who did not receive prenatal care in the DR or did not give birth in the DR were excluded.

Before recruitment, a map of the Barrero community was developed by the study authors led by MJ with the help of community members identified by Misión ILAC, a local non-profit organization and partner of study authors. Using existing Google Maps data, relevant landmarks were identified and missing data was added to create the original, updated, map of Barrero. Study authors (AM, AV, OF) as well as PUCMM students divided this original map of Barrero into 10 zones, incorporating the knowledge of community members; the study authors later walked the Barrero community with community leaders to further refine the map. Community leaders included the Misión ILAC trained and certified community health leader and her peers with geographic and social knowledge of the community.

To recruit participants, study authors AM, AV, and OF walked the Barrero community with one of the three community leaders and collected data using the questionnaire via house-to-house sampling in February 2025. Each day during collection, the study authors AM, AV, and OF requested the community escorts lead them to a different community zone, using the previously created map, to ensure data collection was distributed equally throughout the community. Throughout data collection, the map was marked with each house surveyed to prevent duplicate observations from the same woman. Previous data from Misión ILAC estimated that the Barrero community has approximately 800 families.

The survey was conducted face-to-face with questionnaire items asked orally in Spanish, and after collection, data was anonymized and stored in a secure network drive. Individual consent was obtained from all participants prior to questionnaire administration via an informed consent form. Data collection occurred between February 14, 2025, and February 22, 2025. Dual Institutional Review Board (IRB) approval was obtained from Creighton University Biomedical Institutional Review Board IRB (InfoEd record number: 2005393-01) and Pontificia Universidad Católica Madre y Maestra Institutional Review Board IRB (COBE-FACS-EXT-001-2-2024-2025).

### Rationale for the study design

The design of this study, in which researchers walked the community and spoke directly with women who had given birth was chosen for several reasons. First, in the DR, there is not pervasive use of an electronic medical record system; therefore, the most effective way to collect data for the women of this community was to go to them. Additionally, although there is potential for recall bias, to collect data about women's perceptions and satisfaction, speaking to them directly was necessary. Finally, as this is the first study of its kind in a semi-rural, semi-urban community in the DR, collecting and reporting baseline data is important as it guides future interventions and allows for future data trending to evaluate interventions.

### Study outcomes

The primary outcome of this study was to quantify rates of C-sections in the Barrero community and explore their associations with age, education level, nationality, health insurance type, and delivery facility type. A secondary outcome was developing an understanding of maternal perceptions of why they underwent the chosen delivery method and how these perceptions fit the framework of evidence-based C-section delivery indications (3, 23, 24).

### Data analysis

Quantitative data included both categorial and continuous variables. Categorical variables are presented as frequency count and percentages, whereas continuous variables are presented as median and interquartile range with minimum and maximum values also presented. Delivery rates were evaluated at the pregnancy level with generalized estimating equations (GEE) using compound symmetric working correlation matrix to account for the correlation inherent to mothers providing responses on more than one pregnancy. Differences in C-section rates were compared using a logit link and binomial conditional response distribution (conceptually similar to logistic regression), whereas recovery times were compared using identity link and lognormal conditional response distribution (conceptually similar to lognormal regression). All results reported from the GEE models were unadjusted. Analysis of quantitative data was performed using SAS v. 9.4 with two-tailed *p* < 0.05 used to indicate statistical significance. Open-ended responses were analyzed using thematic analysis of motivations. Two authors, AM and AV, independently grouped factors into subthemes, followed by grouping subthemes into broad thematic categories using an inductive approach. Differences were remedied by discussion with a third author, OF. Themes and subthemes were refined with iterative discussion among AM, AV, and OF, in addition to conversation with senior authors CG and NS, until they were conceptually distinct. Quotes were taken from interviews and translated to English.

## Results

Overall, 121 women aged 18–50 years living in Barrero completed the questionnaire yielding data for 236 pregnancies. Of the 121 women, 110 (91.7%) were Dominican and 10 (8.3%) were Haitian; one participant chose “Prefer not to Respond.” Additional demographic data for participants is shown in more detail in Table 1.

**Table 1:** Demographic characteristics of mothers participating in the survey.

Maternal Characteristics	Frequency count	Percent (%)
Sample size	121	
Nationality, *n* %		
Dominican	110	91.7
Haitian	10	8.3
Employment Status, *n* %		
Unemployed	63	52.1
Formal	31	25.6
Informal	27	22.3
Education, *n* %		
None	4	3.3
Primary	38	31.4
Secondary	60	49.6
University	19	15.7

Most respondents (95.8%) received at least some prenatal care for all of their pregnancies, 86.4% of whom received eight or more prenatal care visits per pregnancy and 81.0% of whom discussed their birth plan during a visit. On average, women traveled 45 min (IQR: 20–60 min) to give birth; however, time traveled ranged from 0 to 180 min. Additional details on pregnancy demographics can be found in Table 2.

**Table 2:** Demographic characteristics of pregnancies measured in the survey.

Pregnancy Characteristics	Frequency count	Percent (%)
Sample Size	236	
Insurance Status, *n* %		
None	69	31.5
Public	33	15.1
Private	117	53.4
Prenatal Care, *n* %		
No	10	4.2
Yes	226	95.8
0	3	1.4
1–3	11	5
4–7	16	7.2
8+	191	86.4
Native Language at Birth, *n* %		
Spanish	220	93.2
Creole	16	6.8

**Figure 1:**
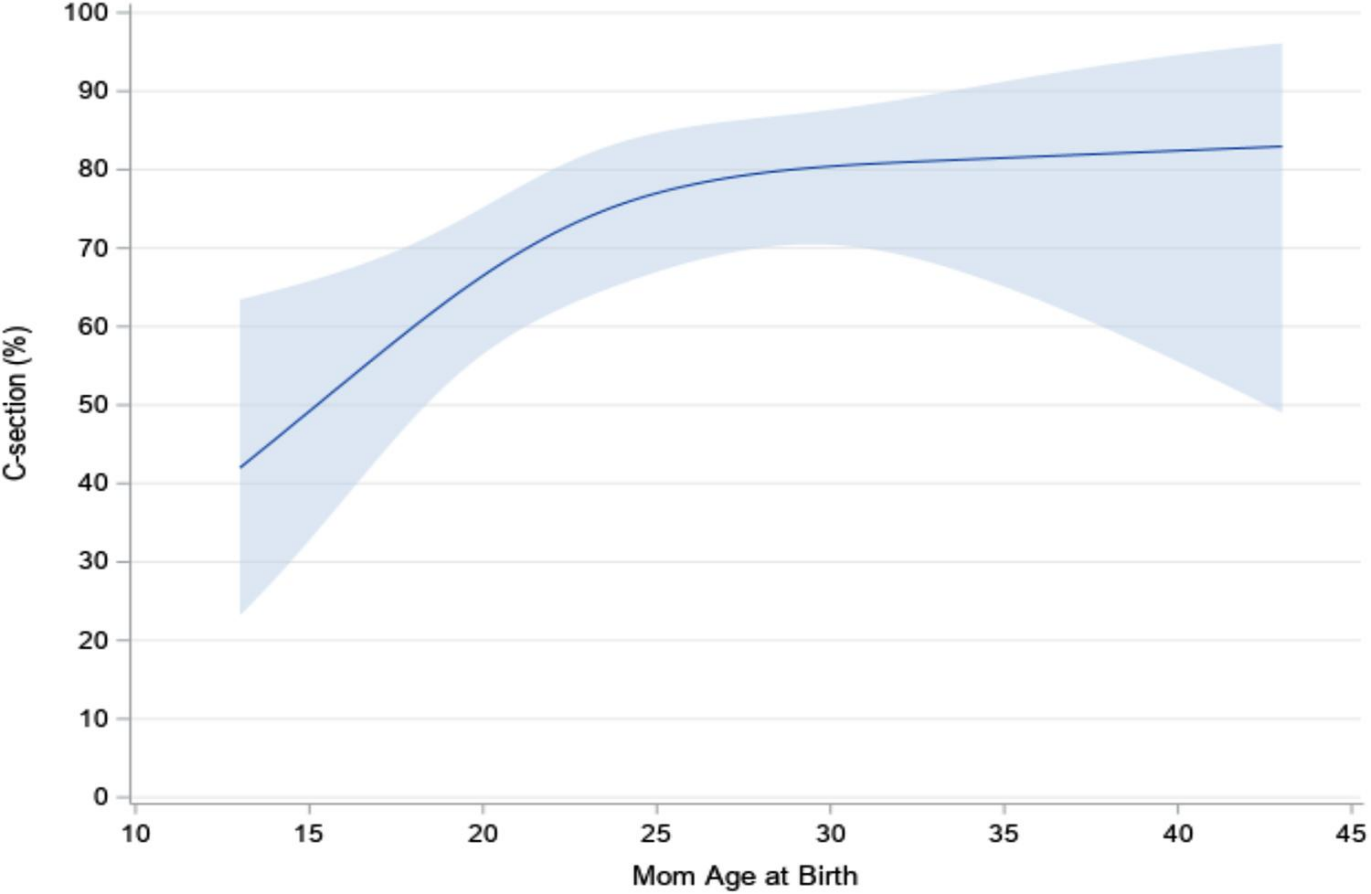
C-Section delivery by Age of mother at time of birth.

Of pregnancies measured, 71.2% were C-sections, of which 61.4% were described as planned (vs. emergent). The overall estimated C-section rate for the Barrero community was 71.5% (95% CI: 63.5% to 78.4%). Women were significantly more likely to undergo C-section if they had any level of education compared to no education (*p* = 0.028), self-identified as Dominican as compared to Haitian (*p* = 0.001), reported private insurance rather than either public or no insurance (*p* = 0.004), and gave birth in a private hospital vs. a public hospital (*p* = 0.013) (Table 3). Older age at delivery positively correlated to C-section rate (Figure 1). No differences in C-section rates were observed by employment status (*p* = 0.341) or receipt of prenatal care (*p* = 0.811) (Table 3).

**Table 3:** Factors associated with C-section delivery.

C-section Rate	Estimate (95% CI)	p
Overall	71.5% (63.5–78.4)	-
Employment		
None	74.0 (62.6–82.9)	0.341
Formal	62.2 (45.7–76.3)	
Informal	76.6 (58.9–88.3)	
Education		
None	16.7 (2.3–63.4)	0.028
Primary	71.6 (60.3–80.8)	
Secondary	69.3 (60.2–77.1)	
University	87.5 (70.9–95.3)	
Insurance Status		
None	56.5 (43.3–68.9)	0.004
Public	65.0 (45.3–80.6)	
Private	82.9 (72.6–89.9)	
Prenatal Care		
No	75.0 (37.5–93.8)	0.811
Yes	71.1 (64.7–76.8)	
1–3	61.3 (16.8–92.6)	0.531
4–7	62.2 (29.5–86.6)	
8+	74.0 (62.9–82.8)	
Nationality		
Dominican	77.2 (69.3–83.6)	0.001
Haitian	9.3 (1.3–45.1)	
Hospital Type		
Public	61.9 (50.6–72.0)	0.013
Private	84.5 (73.2–91.6)	
House	48.2 (3.7–95.8)	

Recovery time, measured in weeks, was significantly longer (*p* = 0.029) for women delivering via C-section, with C-section recovery taking 2.1 (95% CI: 1.7–2.6) weeks and vaginal recovery requiring 1.5 (95% CI: 1.2–2.0) weeks. Of women delivering via C-section, 61.4% described their delivery as planned compared to 39.8% who described theirs as emergent. When asked about motivation regarding delivery method, the majority of women (53.1%) chose “other” as their motivation for planned C-section, whereas another 34.0% were motivated by their provider's preference. In those who underwent emergent C-sections, 90.6% cited “other” as their motivation. Table 4 provides more data on maternal motivation broken down by delivery type.

**Table 4:** Delivery type motivations for All deliveries.

Delivery and Motivation	Frequency count	Percent (%)
C-section	161	71.2
Planned	97	61.4
Motivation Other	51	53.1
Motivation Provider Preference	33	34.0
Motivation My Preference	20	20.6
Motivation Perceived Pain	1	1.0
Motivation Perceived Force	0	0
Motivation Partner Preference	0	0
Motivation Recuperation Time	0	0
Emergent	64	39.8
Motivation Other	58	90.6
Motivation Provider Preference	6	9.4
Motivation Pereived Pain	4	6.3
Motivation Perceived Force	0	0
Motivation Partner Preference	0	0
Motivation My Preference	0	0
Motivation Recuperation Time	0	0
Vaginal Birth	65	28.8
Motivation My Preference	41	65.1
Motivation Other	25	39.7
Motivation Provider Preference	14	22.2
Motivation Perceived Pain	1	1.6
Motivation Perceived Force	0	0
Motivation Partner Preference	0	0
Motivation Recuperation Time	0	0

**Figure 2:**
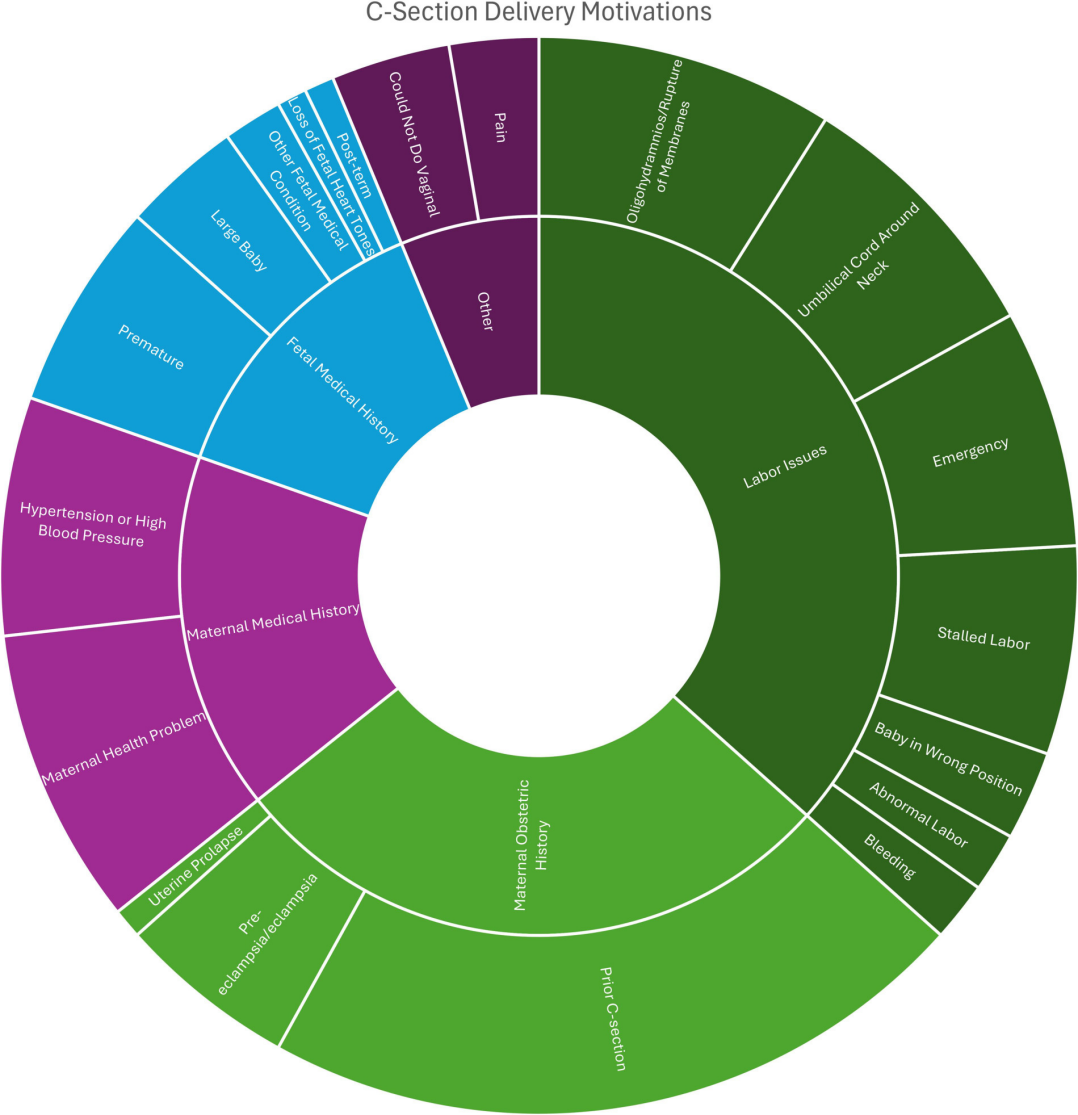
C-Section delivery specific responses to “other”.

For those describing their motivation for any C-section delivery as “other,” five distinct themes emerged: labor issues, maternal obstetric history, maternal medical history, fetal medical history, and other. Each theme encompassed many subthemes. Originally, maternal obstetric history and maternal medical history were grouped together, but through discussion it was decided that these descriptors represented distinct experiences and motivations. Maternal obstetric history included subthemes related to maternal health during her pregnancy while maternal medical history subthemes related to a mother's overall health. Themes, subthemes, and quotes for women delivering via C-section are provided in Figure 2 and Table 5.

**Table 5:** C-Section delivery themes, subthemes, and quotes from “other” category.

Theme	Subtheme	Quotes
Maternal Obstetric History	Prior C-section	"My first was a C-section"
		"I already had one C-section"
		"It was obligatory to do a C-section again"
		"I already had two previous C-sections"
		"It was my preference because my first child was a C-section due to pre-eclampsia"
	Pre-eclampsia/eclampsia	
	Uterine Prolapse	
Maternal Medical History	Maternal Health Problem	"Kidney problem"
		"I had a history of knee surgery"
		"I had a medical need"
		"I had a heart problem"
		"I had a urinary infection at the time of birth"
		"I have asthma"
	Hypertension/High Blood Pressure	
Fetal Medical History	Premature	"Baby was premature"
		"Baby was only 6 months old"
	Large Baby	"Baby was too big"
		"Baby was very big"
		"Baby had a big head"
	Other Fetal Medical Condition	"Baby had a fever"
		"Too much liquid"
	Post-term	"Baby was coming too late"
	Loss of Fetal Heart Tones	
Labor Issues	Abnormal Labor	"Strong contractions"
		"Too much pain for how dilated she was"
	Oligohydramnios/Rupture of Membranes	
	Umbilical Cord Around Neck	
	Emergency	
	Stalled Labor	
	Baby in Wrong Position	
	Bleeding	
Other	Could Not Do Vaginal	"Couldn't feel the contractions"
		"I couldn't do a normal birth”
		"I couldn't push"
	Pain	

**Figure 3:**
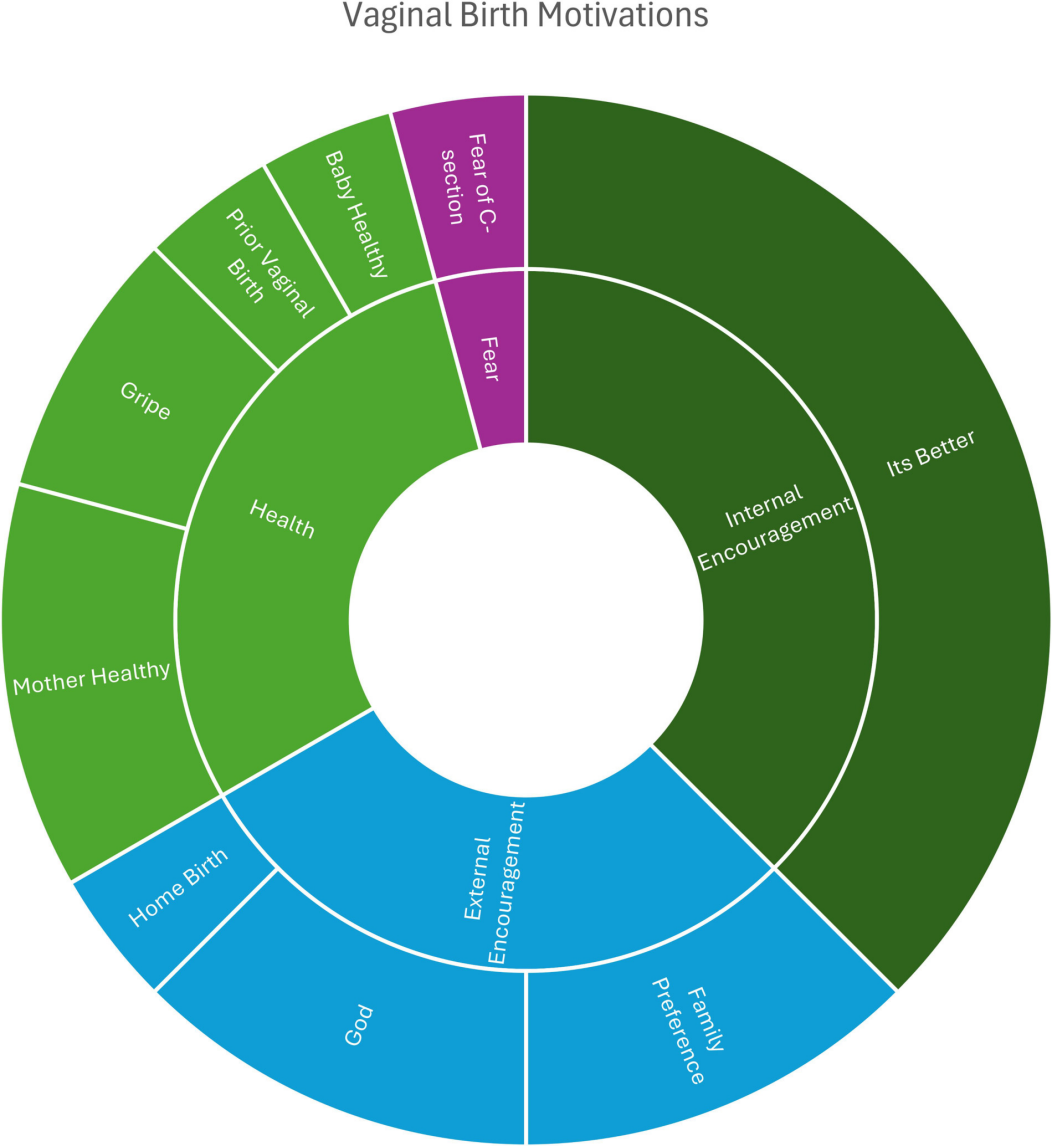
Vaginal delivery specific responses to “other”.

For women who underwent vaginal birth, 65.1% reported they were motivated by their own preference, whereas 22.2% were motivated by their provider's preference, as seen in Table 4. Interestingly, four distinct themes arose from the “other” category for vaginal birth, including internal encouragement, external encouragement, health, and fear. Although health was at first considered by authors as too general, further discussions established that the motivation behind this group of responses was rooted in the mother's perception of her or her baby's health. Figure 3 and Table 6 detail the themes, subthemes, and quotes about motivation for women delivering vaginally.

**Table 6:** Vaginal delivery themes, subthemes, and quotes from “other” category.

Theme	Subtheme	Quotes
Internal Encouragement	Its Better	"It is better"
		"I heard it was better”
		"I always said I would have a normal birth"
		"I did not like the idea of a surgical birth"
External Encouragement	God	"Dios [God]"
	Home Birth	"I gave birth at home"
	Family Preference	
Health	Mother Healthy	"I was healthy"
		"I felt strong in my health"
	The Flu	"Gripe [the flu]"
	Baby Healthy	"My baby was good so I could"
	Prior Vaginal Birth	"I did not have a c-section with my first child"
Fear	Fear of C-section	"I was afraid of a c-section"

## Discussion

According to UNICEF, as of 2019, the national rate of C-sections in the DR was 62.9% (9). This study estimated that the community of Barrero experiences C-section rates of 71.5% (95% CI: 63.5% to 78.4%), higher than the most recent reported national average. However, Hasan et al. calculated trends starting in 1990 and projected through 2018 using Demographic and Health Surveys (DHS) data from the DR that indicated a pattern of an overall rise in C-section rates, with an average annual rate of positive change of 4.2% (12). Therefore, this elevated rate in Barrero could represent a community-specific increase or be reflective of national increases.

Older age at delivery was positively correlated with C-section rate in Barrero, similar to national-level data from the DR. Hasan et al. also investigated the relationship between C-section rates and age and found that older age correlated to rising C-section rates nationally in the DR (12). Notably, one study in Santo Domingo did not find an association between age and emergency C-sections in the DR, indicating the trend may vary based on C-section indication (15). Of the women in our study who described their motivation for a planned C-section as “other,” 41.2% cited a prior C-section as the motivator. Some of the association between older age and higher C-section rate may be a statistical consequence of women delivering multiple children via C-section due to a high rate of first birth being a C-section. Prevalence data looking at delivery types found the DR had the highest prevalence of first birth being cesarean, at 49.1%, compared to the other 58 countries studied (25). Thus, decreasing the primary C-section rate would likely reduce the rate of subsequent C-section delivery for women and should be a priority for healthcare providers and public health interventions in the DR moving forward.

In this study, the C-section rate was higher for all education levels compared with non-education. Researchers found no difference in C-section rates among the groups with any level of education greater than zero. Hasan et al. also identified a pattern of greater C-section rates for women who had completed secondary level education or higher compared with those who had completed less than secondary level education (12). Adu-Bonsaffoh et al., in contrast, did not find education level to have statistically significant odds of increasing emergency C-section rates in the DR (15). Again, C-section indication may play a role in the association, or lack thereof, between education level and C-section delivery.

Migrants from Haiti are the largest ethnic minority group in the DR (26). Although estimates vary, approximately 800,000 people with Haitian ancestry live in the DR, including residents of the DR born in Haiti, people born to Haitian and Dominican parents, and people born to Haitian parents (26–28). Haitian women who have migrated to the DR and Dominican women of Haitian descent experience exclusion and discrimination when receiving healthcare, particularly during pregnancy (29). After recent increased migration restrictions in the DR, the United Nations (UN) identified reports of arrests, detention, and deportation of Haitian migrant women, both pregnant and recently delivered, while attending medical check-ups, which experts noted may deter women from seeking medical assistance (30). Similarly, Dominican-based Observatory for Caribbean Migrants (OBMICA) found that migrant women in the DR face barriers to reproductive health services, as newer migration policies may lead to the discrimination, detention, and deportation of pregnant women (31). They also noted a rise in home births during this time which is believed to be related to fear of deportation (31).

In Barrero, 9.3% of self-described Haitian women delivered via C-section, which was significantly lower than those of self-described Dominican women at 77.2% (32). A paucity of research exists regarding self-described Haitian women delivering in the DR, which may be a result of the discrimination and fear of deportation faced by Haitian girls and women (29–31). However, one small study by Cammett et al. interviewed 62 women from 13 different bateyes in the DR and found that 33.3% (*n* = 19) of Haitian women living in these communities, populated primarily by Haitian migrants and Haitian descendants, delivered via C-section (32). Although the lower rate found in both the previous and present studies may be preferable clinically, it may reflect reluctance to deliver in facilities due to fear of deportation or discrimination rather than clinical indication. According to a case study assessing coverage and inequalities in maternal and child health interventions among Haitian migrants, differences in the proportions of C-section for Haitian women may be explained by the low rates of antenatal care (65.3%) and skilled birth attendance (39.5%), compared to Dominican women (33). C-section delivery requires a skilled birth attendant, so women unable to access the healthcare system due to fear of deportation may not have access to this delivery method. These studies include small sample sizes of Haitian women, making it difficult to correlate assumptions to all Haitian women living in the DR.

In the Barrero community, the C-section rate was significantly higher for those with private insurance compared with either public or no insurance. Although it has not previously been studied in the DR, Hoxha et al. conducted a systematic review and meta-analysis in the United States, Australia, and Ireland to evaluate the association between C-section delivery and private insurance (34). This meta-analysis indicated that privately insured women had 13% higher odds of receiving a C-section compared with women with public insurance. Globally, a well-supported, systemic factor of this association is financial incentivization of C-section delivery (34–39). Examples of such financial incentivization include the higher cost for C-section delivery compared to vaginal leading to higher payouts from private insurance companies and reimbursement arrangements for physicians related to performing a C-section (34, 35, 37). Additionally, hospitals may incentivize physicians to make clinical decisions for patients with private insurance based on potential profit, such as encouraging a C-section due to associated longer hospital stay after delivery (34). Such incentivization has not previously been studied in the DR; however, the correlation between private insurance and higher C-section rate requires future research to evaluate potential physician benefits received in the setting of the DR.

This study found 61.9% of deliveries in public hospitals and 84.5% of deliveries in private facilities were C-sections. Of note, although private facilities provided significantly more C-sections in our study, at 84.5%, the rate in public facilities, 61.9%, also remained considerably high. Two studies in the DR which compared the rate of C-section delivery in public facilities to private facilities found respective differences of 49.0% compared with 86.2% and 32.9% compared with 73.5% (12, 14). In the DR, public hospitals are government-sponsored facilities and may be perceived as providing poorer care whereas private facilities may be perceived as ideal due to high-quality technology and amenities (40, 41). In a global study observing patterns of use and disparities in C-section rates, Boerma et al. found C-section use was 1.6-times more frequent in private facilities with an estimated frequency of 18.3% compared with 11.0% in public facilities (11). This regional and global trend emphasizes the difference in care provided in private and public hospitals, highlighting that perceptions of hospitals may not reflect outcomes. Future studies should explore the factors influencing elevated C-section rates in both private hospital settings and in those with private insurance.

Although one previous study by McLennan in the DR found that prenatal care visits positively correlated with C-section delivery, researchers in this study found no differences in C-section rates in relation to attendance of prenatal care, with a rate for those with no prenatal care of 75.0% compared to those with any prenatal care of 71.1% in Barrero (42). Studies conducted in Nepal and Brazil have found that prenatal care is associated with increased rate of C-sections (20, 43). However, in Shanghai, China, a significant reduction in C-section rates was shown in women who attended prenatal care (44). Notably, in our study women accessed prenatal care for 95.8% of their pregnancies. Prenatal care attendance of at least one visit in the DR is almost universal at 98%, so more nuanced characteristics of the prenatal care visits may have more influence than strictly attendance (45). Due to global and within country variability, further work should explore prenatal care characteristics in the DR.

Interestingly, this study's participants, personal preference was the motivation for 65.1% of vaginal births but only 20.6% of C-section births. Conversely, 22.2% of vaginal births were motivated by provider preference compared to 34% of planned C-section births. In their thesis about performing C-sections in the DR, Rodríguez et al. note that many physicians prefer C-sections because “it is faster compared with vaginal delivery” (46). Another study in Turkey found that some obstetricians held the belief that C-sections were safer than vaginal deliveries (47). Based on motivation data for Barrero, there is a possibility that the provider preference rates for C-section are associated with the time required to perform a C-section or beliefs that C-sections are the safer delivery method, but this is unknown. As such, future investigations should evaluate Dominican healthcare provider beliefs and attitudes toward C-sections to inform future educational interventions for healthcare providers.

This study found, using primary C-section indications established by previous studies for classification, 32 of the 51 women who described their motivation for a planned C-section as “other” fell within acknowledged and common indications for C-section delivery (3, 23, 24). Responses that did not strictly fall into these categories made up 19.6% of the maternal motivations and included such responses as “hypertension or high blood pressure,” medical reasons, “could not do a vaginal birth,” premature delivery, maternal age, and “multiple emergent conditions.” Among those citing medical reasons, one mother reported a history of knee surgery while another was motivated due to asthma. In a United Kingdom (UK) study evaluating maternal-reported motivations for C-section, women also cited medical factors, like concern about hypertension and age; the study indicated women considered C-section to be a prophylactic option to prevent psychological and physical harm (48). A similar study in Iran evaluated cultural beliefs about motivations for C-section, finding themes that women felt C-section was a “prestigious mode of birth” and there was “blind imitation in choosing mode of birth,” with one woman stating “I know and heard that none of the physicians use NVD [natural vaginal delivery] delivery, so cesarean is good” (49). It is possible that a mix of social, cultural, and prophylactic harm prevention may similarly contribute to Dominican women's motivation to deliver via C-section, but future qualitative research is needed to better characterize motivations.

Interestingly, no one interviewed in this study reported perceived recuperation time, perceived force, or partner preference as their motivation for either vaginal or planned C-section delivery. Only one person who delivered vaginally and one who delivered via C-section were motivated by perceived pain. However, maternal perception of recovery time was significantly longer after C-section compared with vaginal delivery, with perceived recovery requiring 2.1 weeks and 1.5 weeks, respectively. This perception is supported by evidence that maternal recovery after vaginal delivery is shorter on average, at about 3 weeks, compared with recovery after C-section, at about 6 weeks (50, 51). During interviews with the women of Barrero, several women stated that they did not have time to spare for recovery. This may indicate a cultural perception regarding recovery after childbirth in the DR.

Notably, women in this study were not asked whether the patient or the physician made the decision of delivery mode, only whether they were motivated by themselves or the physician. Adequate health literacy has been shown to be associated with patient-involved decision making in medically underserved patients, whereas physician-directed decision making is more common in those with low health literacy (52). Although significant work exists to quantify health literacy in Latin America and the Caribbean, there are no studies in the DR that describe health literacy of the general population (53). The variability in maternal description of C-section indication in this study potentially highlights gaps in maternal health literacy and physician-patient education or C-section being performed for reasons outside of typical medical indications.

## Strengths and limitations

A strength of the study was representativeness across Barrero given the decision to cluster the community into 10 zones to spread data collection more evenly throughout the entirety of the community. As Barrero is approximately an 800 family semi-rural, semi-urban community in the DR, results of this study are generalizable to similar communities throughout the country and worldwide. Another strength was having the questionnaire reviewed and edited by two different native Dominican speakers who work with rural Dominican communities to improve questionnaire understandability and decrease bias due to cultural and language differences. Additionally, to our knowledge, this study represents the only data about this topic in a semi-rural, semi-urban community, such as Barrero.

Some limitations of this study include recall bias, sampling bias, and cultural differences. The community chosen for this study was not chosen randomly, rather it was selected by our country partners, Misión ILAC and PUCMM, in the DR. Regarding recall bias, women with children ages 0–12 were chosen to participate to collect extensive data from a population that had not been previously formally studied. Although this may have made it difficult to remember some aspects of care, researchers chose to do this to create a more comprehensive database for this community. The choice to use house-to-house sampling, led by community guides, may have introduced bias due to non-random selection. Although not negligible, researchers chose to partner with community members with lived experience in the community who provided insight on Barrero. A final limitation to the study was that oral interviews with participants were conducted in Spanish. Although interpreters and community guides were part of the data collection team, cultural differences may have influenced information interpretation. No French-Creole interpreter was available to team, so any mothers who spoke only French-Creole were excluded from the study if no other community member was willing and present to assist with Spanish to Creole interpretation.

## Conclusions

C-sections contribute to increased risk of maternal mortality, due to complications like hemorrhage and infection. Identifying factors associated with elevated C-section deliveries help clarify the multifactorial motivations leading to rates beyond clinically indicated levels. In Barrero, DR, C-section was more common among women with any formal education, older maternal age, Dominican nationality, those who had private insurance and those who delivered in private hospitals. While maternal mortality is decreasing worldwide, there remain communities and regions in which it is stagnant or continues to rise. Large cities and rural populations are often studied, but semi-urban, semi-rural communities throughout the DR may be excluded. More work is needed in these communities to gather a broader understanding of country wide patterns and instances in which these communities differ. Future studies in the DR should evaluate interactions among these variables as well as the roles of healthcare institutions, insurance companies, providers, and women to address the rising C-section rate. To help reduce the prevalence of C-sections, community-based interventions to increase health literacy and reproductive agency for women living in the DR, especially semi-rural, semi-urban communities like Barrero, should be developed in collaboration with community leaders.

## Data Availability

The raw data supporting the conclusions of this article will be made available by the authors, without undue reservation.
